# Intractable depression successfully treated with a combination of autogenic training and high-dose antidepressant in department of otorhinolaryngology: a case report

**DOI:** 10.4076/1757-1626-2-6908

**Published:** 2009-08-14

**Authors:** Fumiyuki Goto, Kimiko Nakai, Masato Murakami, Kaoru Ogawa

**Affiliations:** 1 Department of Otorhinolaryngology, Hino Municipal Hospital, Tamadaira 4-3-1, Hino-shi, Tokyo 191-0062, Japan; 2 Department of Otolaryngology, Keio University School of Medicine, 35 Shinanomachi, Shinjuku-ku, Tokyo 160-8520, Japan; 3 Department of Psychosomatic Medicine, Nihon University School of Medicine, Tokyo 160-8520, Japan

## Abstract

**Introduction:**

Patients suffering from ear discomfort are commonly encountered in the department of otolaryngology. If various clinical examinations do not reveal any objective findings, then the patients are referred to the department of internal medicine or psychiatry. Psychotherapy is recommended in some cases. This paper describes the successful administration of autogenic training in a patient suffering from ear discomfort due to major depression.

**Case presentation:**

We present a case of intractable depression that was successfully treated with a combination of psychotherapy, administered by a clinical psychologist, and high-dose antidepressant. The patient was a 36-year-old female with hearing discomfort in her left ear. In 2003, she experienced insomnia and an appetite loss, and her condition was diagnosed as major depression along with an avoidant personality disorder. Her depression has not been improved with antidepressant treatment for 3 years in department of psychosomatic medicine. She was referred to our department because of ear discomfort in her left ear. There was no abnormality in her physical examinations. She wanted to be treated in department of otorhinolaryngology. We increased the dose of fluvoxamine maleate up to 200 mg/day, and introduced cognitive therapy and autogenic training by a clinical psychologist. Eventually, her depressive state as well as the hearing complaint was markedly alleviated.

**Conclusion:**

Autogenic training can be a viable and acceptable treatment option for patients who fail to respond to other therapies. This case emphasizes the importance of autogenic training as a method to control physical symptom of depression.

## Introduction

Patients suffering from ear discomfort are commonly encountered at the otologist in the department of otorhinolaryngology. In the absence of any organic abnormality, such patients are diagnosed with psychological disorders and are often referred to the department of psychosomatic medicine or to a psychologist, because ear discomfort including tinnitus is common complaints to various psychiatric conditions like major depression, somatoform disorder, and anxiety disorders. Psychotherapy including autogenic training (AT) and cognitive behavior therapy (CBT), which can be used for general relaxation and to treat disturbed emotions, is a good treatment option. However, there are no reports on the application of AT to patients with ear discomfort due to major depression in otorhinolaryngology. The present paper describes the successful administration of AT to a patient suffering from ear discomfort intractable to several conventional therapies in department of psychosomatic medicine.

## Case presentation

The patient was a 36-year-old Japanese female with hearing discomfort in the left ear and a feeling of depression. Patient details were as follows: Occupation: housewife; Ethnicity: Japanese. Since 2002, she was being treated as a case of Meniere disease in another hospital. In 2003, she experienced insomnia and an appetite loss, and her condition was diagnosed as major depression along with an avoidant personality disorder in department of psychiatry. She visited department of psychosomatic medicine in another hospital. She was diagnosed as major depression and medical treatment was introduce with fluvoxamine maleate 150 mg (t.i.d.), zolpidem tartrate 5 mg, ethyl loflazepate 0.5 mg and trazodone hydrochloride 25 mg (v.d.s.). She was referred to our department because of the hearing discomfort in the left ear. We could not find out any organic abnormality by physical examinations. We carefully ruled out the potential disorders provoking earfulness including TMJ (temporomandibular joint) dysfunction, and dental infection and so on with the help of dentist who is specialized in oral surgery. There were no tender points to be sufficient to make diagnosis as fibromyalgia. The results of pure tone audiometry indicated normal hearing; hence, the symptom was considered to be a manifestation of depression. She wanted to continue to be treated in our department of otorhinolaryngology; we had started her treatment after informed consent. The results of the psychological examination were as follows: Self-rating Depression Scale (SDS), 52; Japanese version of the Cornell Medical Index (CMI), IV; Manifest Anxiety Scale (MAS), 31, and Y-G (Yatada-Guilford personality inventory); AE. We diagnosed her as major depression by DSM-IV [1] because of her depressed mood most of the day, markedly diminished interest or pleasure in all, significant weight loss, and insomnia.

We decided to focus on treating the patient's anxiety and depression with our clinical psychologist. At the beginning the dose of fluvoxamine maleate was increased up to 200 mg/day and sulpiride 100 mg (b.i.d.). After 1 month following the patient's first visit, a clinical psychologist introduced cognitive therapy and autogenic training (AT). The cognitive therapy focused to improve her low self evaluation. AT was introduced by a clinical psychologist so as to ease her mental stress. The psychotherapy consisted of one 45-minute session every 3 to 4 weeks. The first session began with a brief introduction to the general background information about the cognitive approach, after which the patient was instructed how to perform AT. Thereafter, the patient performed AT in a relaxed sitting position on a chair for 10 minutes 3 times a day. No self-monitoring was advised. The patient was instructed to carry out slow and deep abdominal breathing at the beginning of AT and regular breathing during AT. Autogenic training session was conducted by the following instructions.

The patient was instructed to sit in the meditative posture and scan the body and to imagine the phrase consists from background formula to sixth formula. The background formula is "I am quite relaxed and my mind is extremely calm". The following formula was introduced. The phrase of first standard formula is "my both arms and legs are heavy". The second standard formula is "My both arms and legs are warm" followed by canceling. She diligently and regularly continued this AT routine 3 times a day from background formula to canceling at her home, according to a written timetable. She learned background, first and second standard formulas of AT in 6 psychotherapy sessions. Astonishingly, after the introduction of AT, her mood stabilized and her ear discomfort and insomnia disappeared in a few weeks.

The patient appreciated the effect of AT and admitted a drastic reduction in her distorted cognition. Eventually, her depressive state, anxiety (Table 1) as well as the hearing complaint was markedly alleviated.

**Table 1:** The changes of psychological parameters (SDS and MAS)

Date	Session	SDS	MAS
March	#1	52	31
October	#8	42	23

SDS, Self-rating Depression Scale; MAS, Manifested Anxiety Scale.

## Conclusion

**Figure 1:**
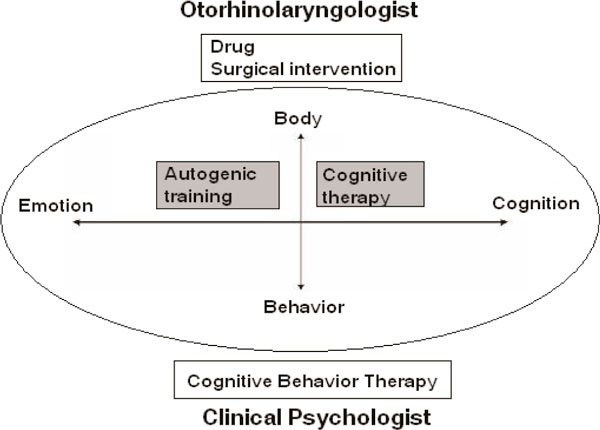
**The role of an otorhinolaryngologist and a clinical psychologist **[7].

As otorhinolaryngologist, we can examine and evaluate physical symptom carefully which is different from psychiatrists. In general the complaints of patients often coincide with their physical findings. However we sometimes encountered the patients whose complaints were not explained by their psychical findings. It is often the case that the depression or anxiety can provoke a variety medically unexplained symptoms. Depression is regularly treated by psychiatrists; however combination of an otorhinolaryngologist and a clinical psychologist can treat the patients with depression complaining psychically unexplained symptoms as in this case. Because an otorhinolaryngologist can focus on physical symptom and a clinical psychologist can focus on behavior and cognition, these two specialists can complementally work (Figure 1).

AT was developed by the German psychiatrist Johannes Schultz and can be achieved by daily self-training sessions of 10 to 15 minutes [2]. AT is a technique for influencing one's autonomic nervous system and it can be used to alleviate many stress-induced psychosomatic disorders. Schultz emphasized parallelism between AT, yoga and meditation. AT has been widely applied as a relaxation technique and has been viewed as a highly effective method for controlling pain and reducing drug dependence substantially [3]. Recently AT was used to treat phobic postural vertigo in department of otorhinolaryngology [4].

We used a psychological approach to treat this patient because her symptoms were closely related to her anxiety and depression and were refractory to conventional therapy. However, AT is not recommended for patients with low-level anxiety, those with little motivation, or those who lack the intellectual capacity to understand and perform AT [5,6]. AT can be a viable and acceptable treatment option for a patient in department of otorhinolaryngology.

## Abbreviations

AT: autogenic training; CBT: cognitive behavior therapy; CMI: Cornell Medical Index; MAS: Manifest Anxiety Scale; SDS: Self-rating Depression Scale; TMJ: temporomandibular joint; Y-G: Yatada-Guilford personality inventory.

## Consent

Written informed consent was obtained from the patient for publication of this case report and accompanying images. A copy of the written consent is available for review by the Editor-in-Chief of this journal.

## Competing interests

The authors declare that they have no competing interests.

## Authors' contributions

All authors read and approved the final manuscript. FG and KN participated in the treatment of the patient and drafted the manuscript. MM and KO provided instructions and advice on the treatment strategy.

## References

[B1] American Psychiatric Association Practice Guideline for Major Depressive Disorder in Adults 2000 Washington D.C: American Psychiatric Association

[B2] Luthe DW SD JH Autogenic Methods 2000 London: The British Autogenic Society

[B3] Kanji N Management of pain through autogenic training Complement Ther Nurs Midwifery 2000 6 143 148 10.1054/ctnm.2000.0473 11858472

[B4] Goto F Nakai K Kunihiro T Ogawa K Phobic postural vertigo treated with autogenic training: a case report Cases J 2008 1 189 10.1186/1757-1626-1-189 18826607 PMC2576179

[B5] Goto F Asama Y Nakai K A case of fibromyalgia treated with medical and autogenic training Nippon Jibiinkoka Gakkai Kaiho 2005 108 1171 1174 16440815 10.3950/jibiinkoka.108.1171

[B6] Goto F Nakai K Kunihiro T Ogawa K Case report: a case of intractable Meniere's disease treated with autogenic training Biopsychosoc Med 2008 2 3 10.1186/1751-0759-2-3 18221543 PMC2265298

[B7] Padesky C Mooney K Presenting the cognitive model to clients Cognitive Therapy Newsletter International 1990 6 13 14

